# A method for estimating Hill function-based dynamic models of gene regulatory networks

**DOI:** 10.1098/rsos.171226

**Published:** 2018-02-21

**Authors:** Faizan Ehsan Elahi, Ammar Hasan

**Affiliations:** National University of Sciences and Technology (NUST), H-12, 44000, Islamabad, Pakistan

**Keywords:** gene regulatory networks, parameter estimation, optimization, *Escherichia coli*

## Abstract

Gene regulatory networks (GRNs) are quite large and complex. To better understand and analyse GRNs, mathematical models are being employed. Different types of models, such as logical, continuous and stochastic models, can be used to describe GRNs. In this paper, we present a new approach to identify continuous models, because they are more suitable for large number of genes and quantitative analysis. One of the most promising techniques for identifying continuous models of GRNs is based on Hill functions and the generalized profiling method (GPM). The advantage of this approach is low computational cost and insensitivity to initial conditions. In the GPM, a constrained nonlinear optimization problem has to be solved that is usually underdetermined. In this paper, we propose a new optimization approach in which we reformulate the optimization problem such that constraints are embedded implicitly in the cost function. Moreover, we propose to split the unknown parameter in two sets based on the structure of Hill functions. These two sets are estimated separately to resolve the issue of the underdetermined problem. As a case study, we apply the proposed technique on the SOS response in *Escherichia coli* and compare the results with the existing literature.

## 1. Introduction

Gene expression is a process that transcribes information of genes and translates it into functional gene products. These products are proteins that play a central role in performing numerous cellular functions. Spatio-temporal expression of these products is controlled by close interaction of different genes with each other, which is commonly known as gene regulatory network (GRN). The large and complex nature of GRNs limits our ability to understand them intuitively [1,2]. Therefore, mathematical models are constructed to get better insight into and understanding of these complex phenomena.

Mathematical models that describe GRNs are of three major types: logical models, stochastic models and continuous models [1]. Logical models [3], e.g. Petri nets, Boolean and Bayesian networks, can only describe qualitative behaviour of GRNs. Stochastic and continuous models, represented by ordinary differential equations (ODEs), can describe the dynamic behaviour of GRNs quantitatively [1,2]. GRNs like any natural system involve randomness and this randomness becomes significant especially when the numbers of molecules are small [4,5]. Single-molecule level models [6] and stochastic differential equations [7] are applied to describe quantitative behaviour with randomness. Stochastic models are complex, computationally expensive and suitable only for a small number of molecules [8]. ODEs, on the other hand, are relatively simple, well studied and can easily simulate the quantitative behaviour of GRNs. These attributes make ODEs preferable whenever randomness is negligible. ODEs are mainly of two types, i.e. linear and nonlinear. Analytical solution of linear ODEs is found easily, but they can be used only in establishing the qualitative behaviour of GRNs [1,9]. GRNs, like most of the physical phenomena, are nonlinear. Therefore, nonlinear ODEs are used in modelling and quantitative analysis of GRNs. Moreover, nonlinear ODEs can easily simulate regulation and feedback effects of genes.

The mathematical model has unknown parameters whose value has to be obtained through experimental data of the biological system. The dynamics of a biological process are captured using time-series experiments that sample the process for measuring the concentration of gene products at different times. These dynamic time-series data can be used for parameter estimation of the ODE model [10]. Data for gene expressions are usually sparse [1,10], i.e. measurements are taken at only a few time points because of limitations of experimental set-up and high expense associated with these experiments. Sparseness of the data forms an underdetermined problem, i.e. the number of equations generated from the experimental data are small compared with the number of unknown parameters of ODEs. Besides sparseness, these measurements suffer from noise that may cause inaccuracies in parameter estimation. Methods for estimating ODE parameters from time-series data can be classified into three broader categories: classical, discretization and collocation methods. Classical techniques are based on first-order Taylor series expansion. First-order expansion for replacing nonlinear structures is only suitable for small durations and mild nonlinearities [11]. Discretization methods, e.g. [12], employ direct numerical solution of ODEs for data fitting. Therefore, ODE parameters are estimated along with initial conditions, which results in an increased number of unknowns. As discretization methods use numerical solutions they are computationally intensive. Moreover, an inherent disadvantage of these methods is that they can result in inaccurate parameter estimation because of high sensitivity with initial conditions [13,14].

Collocation methods [11,15] use basis systems such as polynomials, splines and Fourier basis, etc. Instead of fitting the experimental data directly to the numerical solution of ODEs, data are fitted to a function of basis systems. This function includes coefficients that are determined first and then parameters of ODEs are estimated through these coefficients. Thus, these methods avoid computationally expensive numerical solutions. Another important advantage of these methods is the estimation of initial conditions as a side product of the procedure. On the other hand, solution of nonlinear ODEs is very sensitive to initial conditions and can lead to inaccurate estimates of parameters. The most common variant of collocation methods is the generalized profiling method (GPM) [11]. The GPM employs cascaded optimization. The outer optimization is for parameters of ODEs. The inner optimization is for coefficients of the function of the basis system. The GPM produces better estimates than some of the other variants of collocation methods [15,16]. Detailed discussion of the GPM is given in §2.2

As stated earlier, the optimization problem for GRNs is usually underdetermined because of sparse data [10] and large number of unknowns involved in modelling with ODEs [2]. Moreover, it is a constrained optimization problem due to different reasons such as protein concentration cannot be negative. Nonlinear optimization in collocation methods can be solved by different algorithms. Global optimization algorithms, e.g. particle swarm [17], are very attractive for their convergence to a global solution. However, these algorithms use sampling approach, i.e. they need a large number of function evaluations at each optimization iteration. Function evaluation is computationally expensive in collocation methods because of the cascaded optimization structure. Therefore, algorithms based on a sampling approach are not suitable. Moreover, global optimization algorithms are suitable for a small number of unknowns. Researchers are focusing to improve these two aspects of global optimization algorithms, (e.g. [18,19]). The trust-region-reflective algorithm [20] and the Levenberg–Marquardt algorithm [21] are two other popular techniques of nonlinear optimization. A limitation of the trust-region-reflective algorithm is its inability to deal with underdetermined problems, which is usually the case in GRNs. Levenberg–Marquardt cannot deal with constrained optimization [21].

In this paper, we propose a new optimization approach to solve an underdetermined optimization problem with constraints. This approach is based on Hill functions. Hill functions are considered suitable for building GRN models with ODEs [1,2]. They can quantify activation and inhibition effects of genes. Hill functions are mainly composed of two types of parameters: threshold and cooperativity. Based on the structure of Hill functions, we propose separation of parameters of ODEs into two sets, i.e. threshold and cooperativity parameters. Splitting unknowns into two sets helps in avoiding underdeterminedness. These two sets of parameters are estimated separately with a suitable solver. The two-step estimation is iterated until change in parameters becomes insignificant. Details are given in §3. We take the SOS response in *Escherichia coli* as a case study for the proposed technique and compare the results with work reported in the literature. The case study is discussed in §4.

## 2. Background

**Figure 1.**
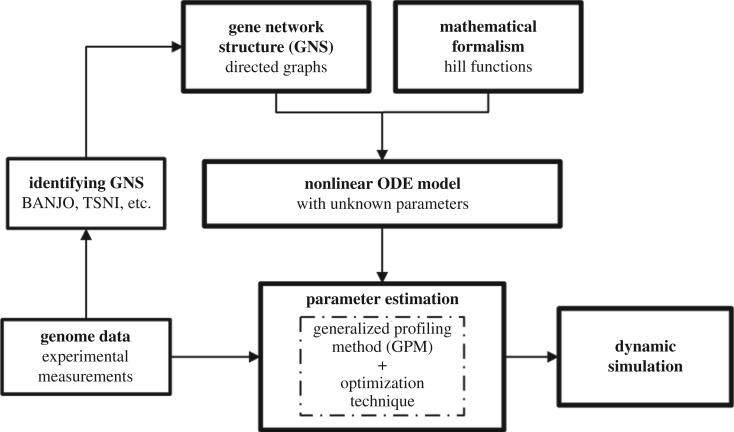
Block diagram describing the estimation of GRNs.

The complete process of parameter estimation of a Hill function-based ODE model from experimental genome data is shown as a block diagram in figure 1. Firstly, gene network structure is obtained either from well-established literature on the subject, experimental observations or by applying reverse engineering techniques (e.g. BANJO [22], ARACNE [23] and TSNI [9]). Network structure is used to construct a Hill function-based ODE model of the GRN with unknown parameters. The parameters of the ODE model are estimated by using the experimental data. Our work is focused on parameter estimation based on the generalized profiling method (GPM) [11]. In the GPM, an optimization problem has to be solved to calculate the most optimal parameters. Lastly, the identified mathematical model can be used for dynamic simulations of the GRN. In the following subsections, we provide details of the Hill function-based modelling and the GPM.

### 2.1 Hill function-based ordinary differential equation model of gene regulatory networks

ODEs belong to the category of continuous mathematical models. Concentrations of gene products are considered as the state variables. The rate of change of these concentrations is expressed as a function of state variables. The standard form is as follows:

2.1 $${\dot{x}}_{i}=f_{i}(x_{1},x_{2},\ldots ,x_{N}),\;i=1,\ldots ,N,$$

where *x*_*i*_ denotes the concentration of the product of gene *i* and *N* is the total number of genes in a GRN. The rate of change of a state ${\dot{x}}_{i}$ is described as some mathematical function *f*_*i*_(.) of all the states. The concentration of any gene product *x*_*i*_ is non-negative. Hence the model should be such that *x*_*i*_≥0 for *i*=1,…,*N* for all practically possible initial conditions [24].

For modelling of GRNs with ODEs, Hill or sigmoidal functions are employed in the literature [25,26]. Hill curves are preferred because they can adopt sigmoidal shape [2,27] with suitable parameters. Mendes [27] has suggested the following function:

2.2 $${\dot{x}}_{i}=P_{i}\prod_{j\in I_{i}}h^{-}(x_{j},Q_{i,j},R_{i,j})\prod_{k\in A_{i}}h^{+}(x_{k},Q_{i,k},R_{i,k})-S_{i}x_{i},\;i=1,\ldots ,N,$$

where

2.3 $$h^{-}(x_{j},Q_{i,j},R_{i,j}):=\frac{{Q_{i,j}}^{R_{i,j}}}{\left({x_{j}}^{R_{i,j}}+Q_{i,j}^{R_{i,j}}\right)}$$

and

**Figure 2.**
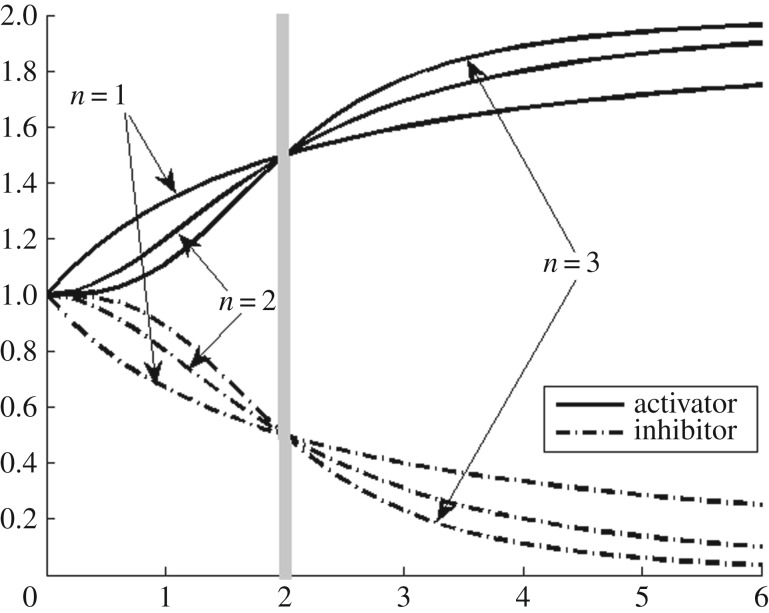
Activator and inhibitor functions are shown for fixed-threshold parameter *Q*_*i*,*j*_=*Q*_*i*,*k*_=2, which is marked by a thick vertical line, while three different curves correspond to three different cooperative parameters *R*_*i*,*j*_=*R*_*i*,*k*_=1,2,3. Increase in the cooperative parameter causes more rapid change across the threshold.

2.4 $$h^{+}(x_{k},Q_{i,k},R_{i,k}):=1+\frac{{x_{k}}^{R_{i,k}}}{\left({x_{k}}^{R_{i,k}}+Q_{i,k}^{R_{i,k}}\right)},$$

where *x*_*i*_ denotes concentration of the *i*th gene product, $I_{i}$ is a subset of {1,…,*N*} that denotes the set of all inhibiting gene products for the *i*th gene, $A_{i}$ denotes the set of all activating gene products for the *i*th gene, *P*_*i*_ is the synthesis rate constant, *S*_*i*_ is the degradation rate constant, *h*^−^ is the inhibiting Hill function, *h*^+^ is the activating Hill function, *Q*_*i*,*j*_ and *Q*_*i*,*k*_ denote the threshold parameters of Hill functions, and the exponents *R*_*i*,*j*_ and *R*_*i*,*k*_ denote the cooperative parameters. Threshold parameter is the value of the concentration after which significant effect of inhibitor or activator is observed. Cooperative parameter controls how sharply the level transition occurs across the threshold. Activator and inhibitor Hill functions are depicted graphically for different values of the cooperative parameter in figure 2.

If the GRN composed of *N* genes has *M* interconnections, then the complete Mendes model of the form (2.2) requires 2*(*M*+*N*) parameters to fully describe the GRN, which includes *N* synthesis rate constants, *N* degradation rate constants, *M* threshold parameters and *M* cooperative parameters . We can write the model (2.2) in compact form as shown below:

2.5 $$\dot{x}(t)=f_{i}(X(t),\theta )\;i=1\ldots N,$$

where *X*=[*x*_1_…*x*_*N*_]^T^ denotes state variables and *Θ*=[*P*_1_…*P*_*N*_ *Q*_1,2_…*Q*_*N*,*N*−1_ *R*_1,2_… *R*_*N*,*N*−1_*S*_1_…*S*_*N*_]^T^ is the vector of all the parameters. The parameters *Θ* have to be estimated from the experimental data.

It may be noted that all the parameters *Θ* should be positive. In a real GRN it is not possible to have negative synthesis rate constants *P*_*i*_ or degradation rate constants *S*_*i*_. Similarly, the concentration thresholds *Q*_*i*,*j*_ or the exponents *R*_*i*,*j*_ of the Hill functions cannot be negative. The necessity of positive parameters should be incorporated in the estimation problem.

### 2.2 Generalized profiling method for estimation of parameters

Estimation is a challenging task because of non-availability of an analytical solution of (2.2) and numerical methods are expensive. Collocation methods avoid these difficulties by using polynomial regression. One of the efficient collocation methods is the generalized profiling method (GPM), which provides accurate estimation with low computational load [11]. In the GPM, β-splines are used as polynomial regression of states and their derivatives. The β-splines are preferred for interpolation as they are differentiable. Further details on β-splines can be found in [28]. The states and their derivatives are defined in terms of β-splines as follows:

$$\begin{array}{rl} x_{i}(t) & =c_{i}^{T}\phi (t)\;i=1\ldots N\\ \;\mathrm{and}\;{\dot{x}}_{i}(t) & =c_{i}^{T}\dot{\phi }(t)\;i=1\ldots N, \end{array}$$

where *c* is column vector of coefficients, while *ϕ*(*t*) denotes β-spline basis systems. Estimation is achieved in two cascaded optimization steps. The inner optimization determines the coefficient vector $c:={\left[c_{1}^{T}\ldots c_{N}^{T}\right]}^{T}$ for a given fixed value of parameters *θ* such that the following objective function is minimized:

2.6 $$\hat{c}(\theta )=\arg\min_{c}\left\{\sum_{t\in T_{E}}\sum_{i=1}^{N}{\left(y_{i}(t)-c_{i}^{T}\phi (t)\right)}^{2}+\sum_{i=1}^{N}\lambda_{i}\int_{t_{0}}^{t_{f}}{\left(c_{i}^{T}\dot{\phi }(t)-f_{i}(c,\phi (t),\theta )\right)}^{2}\;dt\right\},$$

where *y*_*i*_(*t*) is the experimentally observed concentration of the product of the *i*th gene, *t*_0_ is the starting time of the experiment, *t*_*f*_ is the ending time of the experiment, $T_{E}$ is the set of all times at which experimental measurements are available and λ_*i*_ is the weighting parameter. The first term of (2.6) minimizes the sum of squared residuals between the data *y*_*i*_(*t*) and the state of the model $x_{i}(t)=c_{i}^{T}\phi (t)$, whereas the second term penalizes the residuals between derivatives from nonlinear functions *f*_*i*_(.) in (2.5) and their estimates from β-splines. Weighting factor λ_*i*_ is used as a smoothening parameter.

The outer optimization determines the estimate of ODEs parameters $\hat{\Theta }$ by minimizing the following objective function:

2.7 $$\hat{\Theta }=\arg\min_{\theta }\left\{\sum_{t\in T_{E}}\sum_{i=1}^{N}{\left(y_{i}(t)-{\hat{c}}_{i}^{T}(\Theta )\phi (t)\right)}^{2}\right\},$$

where $\hat{c}$ is the optimal value of coefficient vector *c* such that the objective function in (2.6) is minimized. The above-stated objective function is an implicit function of ODE parameters *Θ*. Further details on the GPM can be found in [29].

As mentioned in §2.1, the parameters *Θ* should be positive. Therefore, the above optimization problems are to be solved with the following constraint:

2.8 Θ≥0.

## 3. Proposed method

The nonlinear optimizations in the generalized profiling method in §2.2 have to be solved by numerical optimization algorithms. Having the constraint (2.8) in the optimization problem makes it more complex to solve. Some solvers, such as Lavenberg–Marquardt [21], also do not handle constraints. We propose a reformulation of the mathematical model that allows us to avoid the constraints. Let *P*_*i*_=*e*^*p*_*i*_^, *Q*_*i*,*j*_=*e*^*q*_*i*,*j*_^, *R*_*i*,*j*_=*e*^*r*_*i*,*j*_^ and *S*_*i*_=*e*^*s*_*i*_^. The model (2.2) can be rewritten as follows:

3.1 $${\dot{x}}_{i}=e^{p_{i}}\prod_{j\in I_{i}}h^{-}(x_{j},e^{q_{i,j}},e^{r_{i,j}})\prod_{k\in A_{i}}h^{+}(x_{k},e^{q_{i,k}},e^{r_{i,k}})-e^{s_{i}}x_{i},\;i=1,\ldots ,N.$$

In the above model, the unknown parameters are *θ*=[*p*_1_…*p*_*N*_ *q*_1,2_…*q*_*N*,*N*−1_ *r*_1,2_… *r*_*N*,*N*−1_ *s*_1_…*s*_*N*_]^T^. The benefit of this change of variables is that we do not have to place any constraints on *θ*. Whether the elements of *θ* are negative or positive, the elements of *Θ* will always be positive because of the exponential function.

As mentioned earlier, the number of time samples in experimental data are usually fewer than the number of unknown parameters, i.e. 2(*M*+*N*), in the ODE model (2.2) or (3.1). Therefore, the nonlinear optimizations in the generalized profiling method in §2.2 are underdetermined. This could result in poor estimates of the parameters. Some of the optimization techniques, such as the trust-region-reflective method [20], are more prone to inaccuracy in case of underdeterminedness.

To solve the issue of underdeterminedness, we propose a new approach. The idea is based on the structure of Hill functions. The cooperative parameters *R*_*i*,*j*_=*e*^*r*_*i*,*j*_^ in the Hill functions control the sharpness of the switching action around the threshold parameters. Therefore, cooperative parameters only have prominent impact near the switching of Hill functions. We propose to split the parameters *θ* to be estimated into two sets *θ*_*r*_=[*r*_1,2_…*r*_*N*,*N*−1_]^T^, which includes only the cooperative parameters *r*_*i*,*j*_, and *θ*_*p*_=[*p*_1_…*p*_*N*_ *q*_1,2_…*q*_*N*.*N*−1_ *s*_1_…*s*_*N*_]^T^, which includes all the rest of the unknown parameters. We propose to initially assume a value of *θ*_*r*_, e.g. 2 for all *r*_*i*,*j*_, and estimate only the parameters *θ*_*p*_. As the number of unknown parameters are reduced, it helps to improve the issue of underdeterminedness. However, as *θ*_*r*_ was initially guessed, we then fix the value of *θ*_*p*_ and solve the optimization problem to estimate *θ*_*r*_. We repeat this process until the estimates of all the parameters converge to a value.

**Figure 3.**
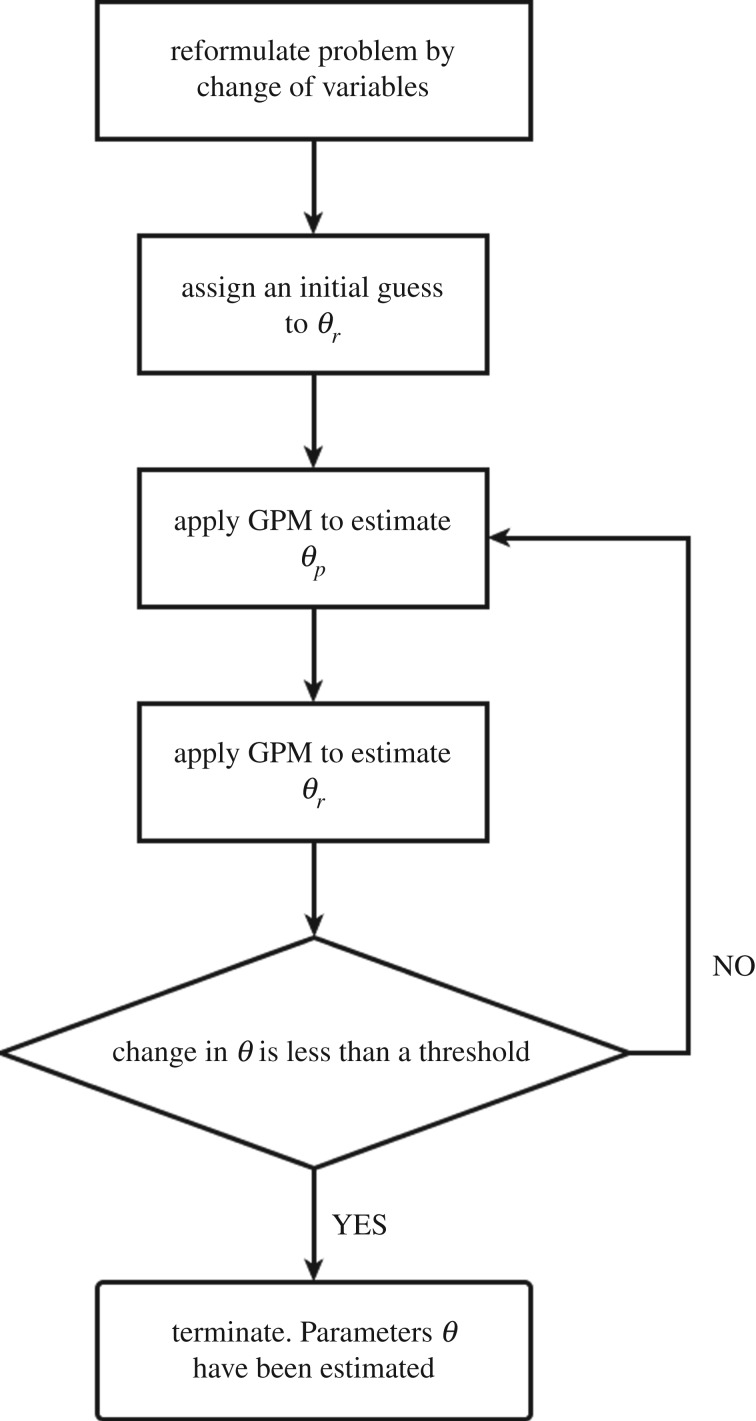
Flow chart for the proposed scheme.

The complete proposed scheme is illustrated with a flow chart in figure 3. Initially the estimation problem is reformulated with the change of variables to avoid constraints. An initial value of *θ*_*r*_ is assumed. The GPM method is applied to only estimate the parameters *θ*_*p*_, which has *M*+2*N* elements. Using the recent optimal values of *θ*_*p*_, the GPM method is applied to only estimate the parameters *θ*_*r*_, which has *M* elements. If the change in all the parameters *θ* is less than some threshold, then the estimation process is complete; otherwise repeat the process.

## 4. Case study: SOS response in *Escherichia* *coli*

**Figure 4.**
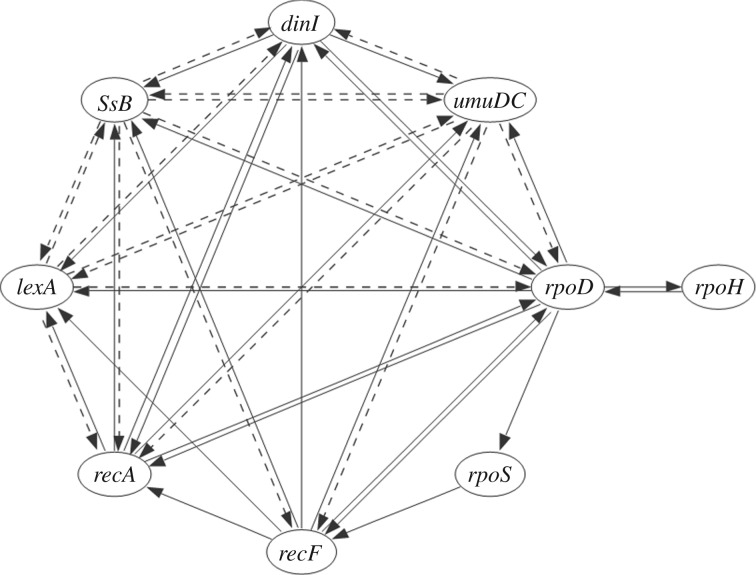
Network interconnection of nine genes SOS response in *Escherichia coli*. Solid lines denote activating interactions, dotted lines denote inhibiting interactions and arrow heads show the direction of effect. Reproduced with permission from [9,30].

SOS response in *Escherichia coli* is an inducible DNA repair system that enables bacteria to survive under severe DNA damage. In the SOS response, nearly 40 genes are directly regulated by *recA* and *lexA*, while tens of other genes are indirectly controlled [30]. Nine genes named *lexA*, *recA*, *recF*, *rpoD*, *rpoS*, *dinI*, *umuDC*, *rpoH* and *ssB* are considered to be directly participating in the SOS response. Established network connections among these nine genes are known in the literature [9,30,31]. Network structure of these nine genes is shown as in figure 4.

### 4.1. Hill function-based model of the SOS response

The ODE model of the SOS response in *Escherichia coli* is constructed based on the network structure given in figure 4 [9,30]. The network has nine genes and 43 interconnections, i.e. *N*=9 and *M*=43. The Hill function-based ODEs for the nine gene product concentrations are given below:

$$\begin{array}{rl} \dot{lexA} & =P_{1}*h^{+}(recA,Q_{1},R_{1})*h^{+}(recF,Q_{2},R_{2})*h^{+}(rpoD,Q_{3},R_{3})\\ & \;*h^{+}(dinI,Q_{4},R_{4})*h^{-}(ssB,Q_{5},R_{5})*h^{-}(umuDC,Q_{6},R_{6})-S_{1}*lexA,\\ \dot{recA} & =P_{2}*h^{+}(recF,Q_{7},R_{7})*h^{+}(rpoD,Q_{8},R_{8})*h^{+}(dinI,Q_{9},R_{9})\\ & \;*h^{-}(lexA,Q_{10},R_{10})*h^{-}(ssB,Q_{11},R_{11})*h^{-}(umuDC,Q_{12},R_{12})-S_{2}*recA,\\ \dot{recF} & =P_{3}*h^{+}(rpoD,Q_{13},R_{13})*h^{+}(rpoS,Q_{14},R_{14})*h^{-}(ssB,Q_{15},R_{15})\\ & \;*h^{-}(umuDC,Q_{16},R_{16})-S_{3}*recF,\\ \dot{rpoS} & =P_{4}*h^{+}(rpoD,Q_{17},R_{17})-S_{4}*rpoS,\\ \dot{rpoD} & =P_{5}*h^{+}(recF,Q_{18},R_{18})*h^{+}(recA,Q_{19},R_{19})*h^{+}(dinI,Q_{20},R_{20})\\ & \;*h^{+}(rpoH,Q_{21},R_{21})*h^{-}(ssB,Q_{22},R_{22})*h^{-}(lexA,Q_{23},R_{23})\\ & \;*h^{-}(umuDC,Q_{24},R_{24})-S_{5}*rpoD,\\ \dot{umuDC} & =P_{6}*h^{+}(rpoD,Q_{25},R_{25})*h^{+}(recF,Q_{26},R_{26})*h^{+}(recA,Q_{27},R_{27})\\ & \;*h^{-}(lexA,Q_{28},R_{28})*h^{-}(dinI,Q_{29},R_{29})*h^{-}(ssB,Q_{30},R_{30})-S_{6}*umuDC,\\ \dot{dinI} & =P_{7}*h^{+}(rpoD,Q_{31},R_{31})*h^{+}(recF,Q_{32},R_{32})*h^{+}(recA,Q_{33},R_{33})\\ & \;*h^{-}(lexA,Q_{34},R_{34})*h^{-}(ssB,Q_{35},R_{35})*h^{-}(umuDC,Q_{36},R_{36})-S_{7}*dinI,\\ \dot{ssB} & =P_{8}*h^{+}(dinI,Q_{37},R_{37})*h^{+}(rpoD,Q_{38},R_{38})\\ & \;*h^{+}(recF,Q_{39},R_{39})*h^{+}(recA,Q_{40},R_{40})\\ & \;*h^{-}(umuDC,Q_{41},R_{41})*h^{-}(lexA,Q_{42},R_{42})-S_{8}*ssB,\\ \dot{rpoH} & =P_{9}*h^{+}(rpoD,Q_{43},R_{43})-S_{9}*rpoH. \end{array}$$

A total of 2*(43+9)=104 parameters are needed to fully describe the above model. We can write the above model in compact form as shown below:

4.1 $${\dot{x}}_{i}=f_{i}(X(t),\theta )\;i=1\ldots 9,$$

where *X*:=[*x*_1_…*x*_9_]^T^= [*lexA* *recA* *recF* *rpoS* *rpoD* *umuD* *dinI* *ssB* *rpoH*]^T^ denotes state variables, and *Θ*=[*P*_1_…*P*_9_ *Q*_1_…*Q*_43_ *R*_1_…*R*_43_ *S*_1_…*S*_9_]^T^ is the set of unknown parameters. The value of *Θ* has to be estimated from the experimental genome data.

### 4.2. Results of the proposed method

In this section, the results of estimation by the proposed method are presented and compared with the results reported in the literature. SOS is a well-studied response, and a lot of literature and experimental datasets are available. Many Microbe Microarrays Database M3D [32] provides datasets for the SOS response under different perturbations, like ultraviolet light or some antibiotic. M3D database provides datasets in two formats, i.e. time course and steady-state measurements. Both forms of data are employed in this work. Time course measurements have been used for estimation of the model parameters. Experimental measurements of steady-state values have been used for validation.

Parameters are estimated with an initial guess of *r*_*i*,*j*_=2. For the GPM, the weighting factor λ_*i*_ is chosen as 150 for *i*=1,…,*N*. The basis system is chosen as β-splines of order 4 with 100 knots, which is the number of joints in the spline function. The choice of λ_*i*_, order and knots is subjective and chosen based on the required smoothness. The algorithm converged in four iterations of the loop in figure 3 with a total execution time of approximately 82 minutes on a PC with a Core i5-650 processor. The Matlab code is available at [33]. Estimated parameters are given in table 1.

**Table 1.** SOS response model parameters estimated by the proposed scheme.

parameter	estimated value	parameter	estimated value
*P*_1_	0.82645	*S*_1_	0.56596
*P*_2_	3.0312	*S*_2_	0.06717
*P*_3_	26.844	*S*_3_	1.151
*P*_4_	0.22028	*S*_4_	0.03181
*P*_5_	22.807	*S*_5_	0.97227
*P*_6_	30.438	*S*_6_	0.037697
*P*_7_	5.7443	*S*_7_	0.024872
*P*_8_	9.6769	*S*_8_	0.60515
*P*_9_	1809.6	*S*_9_	258.52
*Q*_1_	1.6273	*R*_1_	1.6354
*Q*_2_	0.58209	*R*_2_	1.6833
*Q*_3_	0.65218	*R*_3_	1.8982
*Q*_4_	14.988	*R*_4_	2.3757
*Q*_5_	17.705	*R*_5_	2.2303
*Q*_6_	272.67	*R*_6_	1.5392
*Q*_7_	11.981	*R*_7_	2.674
*Q*_8_	56.946	*R*_8_	3.5452
*Q*_9_	4.9391	*R*_9_	1.4626
*Q*_10_	34.691	*R*_10_	1.3153
*Q*_11_	4.779	*R*_11_	1.8499
*Q*_12_	18.819	*R*_12_	3.0102
*Q*_13_	61.511	*R*_13_	4.9999
*Q*_14_	1.3297	*R*_14_	1.1567
*Q*_15_	8.4207	*R*_15_	1.8104
*Q*_16_	11.72	*R*_16_	2.117
*Q*_17_	14.013	*R*_17_	2.049
*Q*_18_	11.867	*R*_18_	1.8588
*Q*_19_	9.5381	*R*_19_	2.2855
*Q*_20_	1.7108	*R*_20_	3.3169
*Q*_21_	4.6155	*R*_21_	2.3944
*Q*_22_	23.779	*R*_22_	2.9639
*Q*_23_	39.778	*R*_23_	1.7128
*Q*_24_	3.0976	*R*_24_	2.0704
*Q*_25_	1.5401	*R*_25_	0.60901
*Q*_26_	0.9762	*R*_26_	1.3191
*Q*_27_	13.822	*R*_27_	4.0316
*Q*_28_	1.3686*10^6^	*R*_28_	1.8207
*Q*_29_	0.50903	*R*_29_	1.9367
*Q*_30_	150.6	*R*_30_	1.3081
*Q*_31_	9.3893	*R*_31_	1.9267
*Q*_32_	134.06	*R*_32_	1.9118
*Q*_33_	15.778	*R*_33_	2.3397
*Q*_34_	2.4818	*R*_34_	1.9367
*Q*_35_	8.9587	*R*_35_	1.7976
*Q*_36_	40.718	*R*_36_	1.3055
*Q*_37_	14.545	*R*_37_	2.6053
*Q*_38_	376.12	*R*_38_	1.1819
*Q*_39_	351.21	*R*_39_	1.8328
*Q*_40_	27.191	*R*_40_	1.9916
*Q*_41_	133.91	*R*_41_	2.9068
*Q*_42_	9.7889	*R*_42_	2.0699
*Q*_43_	14.225	*R*_43_	2.041

**Figure 5.**
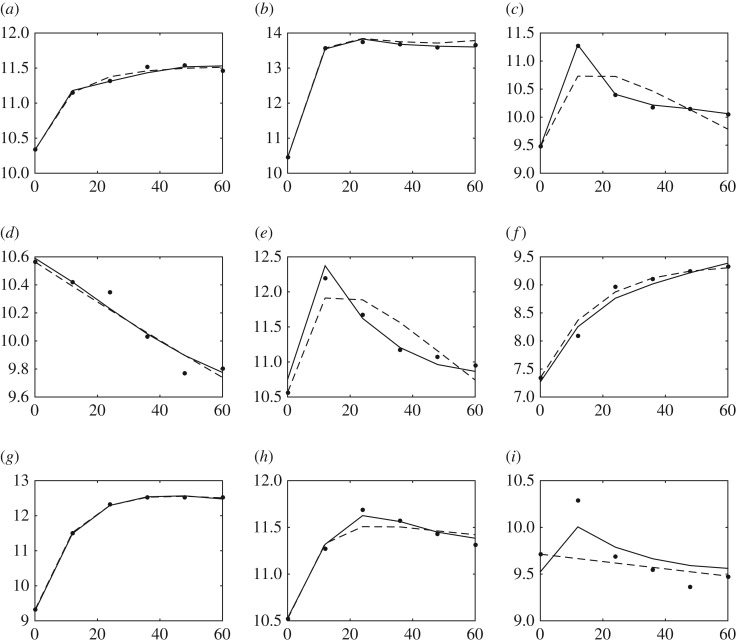
Comparison of experimental and simulated data concentration of gene products: experimental (dots), simulation of the model estimated by the proposed method (solid lines) and simulation of the model estimated in [31] (dashed lines). All the *x*-axes are in time (s) and all the *y*-axes are in concentration (mM). (*a*) *lexA*, (*b*) *recA*, (*c*) *recF*, (*d*) *rpoS*, (*e*) *rpoD*, (*f*) *umuD*, (*g*) *dinI*, (*h*) *ssB* and (*i*) *rpoH*.

The estimated model is simulated to generate time course evolution of gene product concentrations. The results are compared with experimental data and the model reported in [31]. Baralla *et al.* [31] also investigated the same subpart of the SOS response using the same dataset of M3D. They have applied the particle swarm algorithm for optimization and a direct numerical solution of the ODE model for data fitting. It can be seen in figure 5 that dynamic simulation with the model estimated by the proposed method is much more accurate than that of [31], especially for *recF*, *rpoD*, *ssB* and *rpoH*.

For the purpose of validation, the model estimated by the proposed method has also been used to calculate the steady-state values of concentrations. The steady-state values are given in table 2 along with the experimentally observed values and the values obtained by the estimated model in [31]. The table also gives the sum of squared error between the experimental data and values obtained from estimated models. It can be seen that the total error in the values obtained by the model estimated by the proposed method is much smaller than that of [31].

**Table 2.** Comparison of steady-state values obtained by the model estimated by the proposed method and the model estimated in [31].

gene	experimental (mM)	values obtained by the proposed method (mM)	values obtained by [31] (mM)
*lexA*	11.471	11.192	11.4599
*recA*	11.795	13.326	16.492
*recF*	8.975	9.8327	4.87727
*rpoS*	10.383	9.6427	3.46706
*rpoD*	9.4618	11.322	5.20705
*umuD*	7.8192	9.6932	9.35587
*dinI*	9.9169	11.542	12.1365
*ssB*	10.213	11.334	11.2432
*rpoH*	8.5983	9.6991	1.12474
sum of squared errors	—	15.787	169.03

## 5. Conclusion

Obtaining a mathematical model of GRNs requires estimation of model parameters from the experimental data. To estimate the optimal values of parameters an optimization problem has to be solved. Usually the experimental data have much fewer measurements than the number of parameters which could result in an underdetermined optimization problem. Moreover, depending on how the optimization problem is posed, constraints have to be incorporated to find practically feasible values of parameters, which could result in numerical issues. In this paper, we have proposed a new approach to reformulate the estimation problem such that constraints are not required. Based on the structure of Hill functions, we also suggest to split the number of unknowns into two sets, which are estimated one at a time. This helps to alleviate the issue of underdeterminedness.

The proposed technique is applied to estimate the model of the SOS response in *Escherichia coli*. The results are compared with the existing literature and experimental data. Both dynamic and steady-state results show that the proposed technique provides more accurate estimates of the unknown model parameters.

## Data accessibility

The Matlab code of the proposed algorithm is available from the Dryad Digital Repository: https://doi.org/10.5061/dryad.ht047 [33].
